# The Protective Effects of *Securigera securidaca* Seed Extract on Liver Injury Induced by Bile Duct Ligation in Rats

**DOI:** 10.1155/2022/6989963

**Published:** 2022-02-04

**Authors:** Zahra Nasehi, Nejat Kheiripour, Maryam Akhavan Taheri, Abolfazl Ardjmand, Faezeh Jozi, Esmat Aghadavod, Amir Hossein Doustimotlagh, Mohammad Esmaeil Shahaboddin

**Affiliations:** ^1^Institute for Basic Sciences, Research Center for Biochemistry and Nutrition in Metabolic Diseases, Kashan University of Medical Sciences, Kashan, Iran; ^2^Institute for Basic Sciences, Anatomical Sciences Research Center, Kashan University of Medical Sciences, Kashan, Iran; ^3^Institute for Basic Sciences, Physiology Research Center, Kashan University of Medical Sciences, Kashan, Iran; ^4^Department of Clinical Biochemistry, Faculty of Medicine, Kashan University of Medical Sciences, Kashan, Iran; ^5^Medicinal Plants Research Center, Yasuj University of Medical Sciences, Yasuj, Iran

## Abstract

This study is aimed at evaluating the effects of *Securigera securidaca* (SS) seed extract on cholestatic liver injury induced by bile duct ligation (BDL) in rats. Total polyphenols and flavonoids in SS seed extract were determined using a colorimetric assay, and their components were quantified using HPLC. Rats in four groups underwent BDL at the common bile duct and were treated for 21 days with either oral distilled water as vehicle, vitamin C, 100 mg/kg SS seed extract, or 200 mg/kg SS seed extract. Rats in the fifth group underwent abdominal incision without BDL and were treated with distilled water, and rats in the sixth group were healthy and received nothing. Finally, rats were sacrificed, blood samples were analyzed through biochemical methods, liver tissues were histologically assessed, and the expression of the TGF*β*-1, iNOS, caspase-3, and *α*-SMA genes in the liver was assessed through real-time PCR. BDL significantly increased, and SS seed extract significantly decreased the serum levels of bilirubin and liver function enzymes. Moreover, SS seed extract suppressed the expression of the TGF*β*-1, iNOS, caspase-3, and *α*-SMA genes, reduced the levels of nitric oxide, malondialdehyde, and protein carbonyl, and increased the levels of glutathione, total antioxidant capacity, and SOD and catalase enzyme activity in the serum and liver. Extract at a dose of 100 mg/kg had significant positive effects on liver morphology and parenchyma structure in a dose-dependent manner.

## 1. Introduction

Cholestasis is a prevalent liver problem caused by the acquired or congenital obstruction of the bile ducts. Prescription drugs, over-the-counter drugs, and herbal remedies are the most common causes of acute cholestasis in adults, which may sometimes progress to a chronic vanishing bile duct syndrome [1]. The accumulation of highly toxic bile acids in the liver causes cholestatic liver injury, hepatitis, proliferation of hepatic stellate cells, collagen synthesis, liver fibrosis (LF), cirrhosis, and death [2]. Several LF screening studies on the general public reported that the prevalence of LF varied from 2% to 27% [3–6]. LF prevalence was also 3.6% in a study in Europe and 2.9% in a study in South Korea [7]. Moreover, a study in Iran showed that the prevalence of advanced LF among obese people was 10.4% [8].

The exact mechanism of LF is still poorly known. However, oxidative stress and inflammation seem to be two major contributing factors [9–11]. In chronic oxidative stress and inflammation, inflammatory cytokines and fibrogenic growth factors stimulate fibroblasts and hepatic stellate cells to differentiate into myofibroblasts and activated hepatic stellate cells, respectively. These cells express alpha-smooth muscle actin and lead to the formation of a collagen-rich extracellular matrix. Moreover, the fibrogenic cytokine transforming growth factor-beta (TGF*β*) plays a significant role in the development of LF and cirrhosis through stimulating matrix protein production and inhibiting matrix protein elimination [12, 13]. On the other hand, the accumulation of bile acids during cholestasis negatively affects mitochondria and thereby, induces oxidative stress and apoptosis of hepatocytes [14].

Given the critical role of oxidative stress and inflammation in the pathogenesis of liver diseases, agents which modulate oxidative stress and inflammation can be used to prevent and manage these diseases. Medicinal plants, such as *Securigera securidaca* (SS), are among these agents. SS is an herbaceous plant from the *Fabaceae* family and is known with common names such as ax, hatchet vetch, scorpion vetch, weed seed, and goat pea [15]. It is widely distributed in different areas of Europe, Australia, and Asia and is also found in Iran [16]. SS is a plant with wide use in eastern traditional medicines, such as the traditional medicines of India, Egypt, and Iran, for health problems such as epilepsy, hypertension, malaria, gastric influx, and hyperlipidemia. Phytochemical analysis of SS shows that SS seed has active biological compounds such as phenols, flavonoids, saponins, tannins, and high levels of unsaturated fatty acids [17–19]. A study into the effects of SS detected that SS includes 47 phenolic compounds in sixteen categories, namely, eight phenolic acids (such as the derivatives of hydroxybenzoic and hydroxycinnamic acids) and 39 flavonoids [20].

Recent studies in animal models showed that herbal phenols and polyphenols, particularly flavonoids, are effective in reducing triglycerides, oxidative stress, and lipid peroxidation [21]. Flavonoids are herbal antioxidants with antifibrotic and anticancerogenic effects and, hence, can suppress LF. Moreover, flavonoids affect the signaling of nuclear factor kappa-light-chain-enhancer of activated B cells (NF-*κ*B) and tumor necrotizing factor alpha, prevent the accumulation of extracellular matrix protein through promoting the apoptosis of activated hepatic stellate cells, and thereby, exert antifibrotic effects [22]. Moreover, a study reported that SS seed extract reduced the effects of oxidative factors such as malondialdehyde (MDA) and promoted the expression of antioxidant enzyme superoxide dismutase (SOD) [21]. Another study on rats with streptozotocin-induced diabetes mellitus revealed that twice-daily administration of 500 mg/kg SS seed extract (which included methanolic and chloroformic fractions with a 70 : 30 ratio) for two weeks reduced hepatocyte injury [17].

To the best of our knowledge, none of the previous studies evaluated the effects of SS on cholestatic liver injury. Therefore, the present study was conducted to narrow this gap. The aim of the study was to evaluate the effects of SS seed extract on cholestatic liver injury in rats induced by bile duct ligation (BDL).

## 2. Materials and Methods

### 2.1. Chemicals and Reagents

Trichloroacetic acid (TCA), 5,5-dithionitrobenzoic acid (DTNB), thiobarbituric acid (TBA), isopropanol, p-dimethylamino benzaldehyde, 2,4-dinitrophenylhydrazine (DNPH), *meta*-phosphoric acid, 2-amino-2-hydroxymethyl-propane-1,3-diol-hydrochloride (Tris—HCl), glacial acetic acid, guanidine hydrochloride, phenylmethylsulfonyl fluoride (PMSF), bovine serum albumin (BSA), diethyl pyrocarbonate (DEPC), chloroform, boric acid, and ammonium molybdate were obtained from Kalazist Co. (Tehran, Iran). Ascorbic acid, vanadium chloride, sodium nitrite, ethylenediamine tetra-acetic acid (EDTA), Tris base, tetra ethoxy propane (TEP), N-(l-naphthyl)-ethylendiamine-dihydrochloride (NEDD), sulfanilamide, sodium acetate, ethyl acetate, 4-(2-hydroxyethyl)-1-piperazineethanesulfonic acid (HEPES), dithiothreitol (DTT), Coomassie blue, hydrogen peroxide, and Triton X-100, were obtained from Merck (Darmstadt, Germany). Ethanol, formalin, and hydrochloric acid (HCl) were obtained from Mojallali Co. (Tehran, Iran). 2,4,6-Tripyridyl-S-triazine (TPTZ) was obtained from Sigma-Aldrich (Missouri, US).

### 2.2. Preparation of SS Seed Extract

SS seeds were obtained from naturally grown SS plants during spring in Abadeh, Fars province, Iran, and were recognized by the Faculty of Pharmacy of Tehran University of Medical Sciences, Tehran, Iran. Some seeds were kept as reference in the herbarium of the Pharmacognosy Department of this faculty (reference number: PMP-1702). Seeds were dried in shade and completely powdered using a blender. Then, 500 grams of the powder was soaked in 1000 milliliters of 80% ethanol, and the mixture was slightly shaken for 72 hours at room temperature in a dark room. After that, the extract was filtered and concentrated at a temperature of 45 ± 1°C and low pressure using a rotator evaporator. Accordingly, a concentrated extract with a bright brown color was obtained which was dried at room temperature and powdered. The weight of the powder was 38.5 grams (i.e., a yield of 7.7%).

### 2.3. High-Performance Liquid Chromatography (HPLC) Analysis

The total phenolic and flavonoid content of SS seed extract was determined according to the guideline of European Pharmacopoeia (EP 8.0, 2.2.25). Total polyphenols were expressed as mg gallic acid equivalents per gram dry weight (mg GASE/g D.W.), and total flavonoids were expressed as mg quercetin equivalent (QE) per gram dry weight. Total polyphenols and flavonoids in SS seed extract were determined using a colorimetric assay, and their components were quantified using HPLC. HPLC analysis was performed using the KNAUER AZURA HPLC system (Berlin, Germany) with a Teknokroma reversed-phase 300SB C18 column (250 millimeters) and particle size of five millimeters (Lawrence, KS, USA) as well as an ultraviolet detector set at 340 nanometers. Sample and authentic standards (50 mL; luteolin, apigenin 7-glycoside, and isorhamnetin) were dissolved in dimethyl sulfoxide, acidified using citric acid, and injected into the column. The mobile phase consisted of 0.4% formic acid and acetonitrile (60 : 40, *v*/*v*) with a constant flow of one milliliter per minute. Isolated peaks from the phenolic compounds in the sample were identified through comparing their relative retention time with standard values. Finally, the concentration of each compound was determined through calculating peak area integration and comparing it with standards.

### 2.4. Design

This experimental study was conducted in 2020 in the Clinical Biochemistry Department and the Physiology Research Center of Kashan University of Medical Sciences, Kashan, Iran.

### 2.5. Animals and Intervention

Forty-eight adult male Wistar rats (200–250 grams) were housed in a controlled animal center with a temperature of 25 ± 0.5°C, humidity of 50%–70%, 12/12 light/dark cycles (07:00–19:00), and free access to food and water. They were randomly assigned to six eight-rat groups. Under general anesthesia, rats in four groups underwent BDL at the common bile duct and then, were treated with either distilled water as vehicle (BDL-control group), 4.25 *μ*g/kg vitamin C as an agent with known antioxidant effects (BDL+VitC group), 100 mg/kg SS seed extract (BDL+SS100 group), or 200 mg/kg SS seed extract (BDL+SS200 group). The LD50 of SS ethanolic extract was found to be 2 g/kg [20]. The effective dose of 100 and 200 mg/kg was selected based on LD50 of plant and our previous study [19]. Similarly, L-ascorbic acid (4.25 g/mL) as a positive reference antioxidant was selected based on previous studies [20] and is considered a low dose. Rats in the sham group underwent abdominal incision without BDL and were treated with distilled water as vehicle. Rats in the sixth group were healthy and received nothing (healthy group). Vehicle, vitamin C, and SS seed extract were orally administered once daily through gastric gavage. The three-week course of treatment was initiated 48 hours post the BDL procedure. Prior to blood sampling, the rats were anesthetized with diethyl ether to ease handling, and blood samples were collected through cardiac puncture using a 25 G, 1^″^ needle. After as much blood as possible was withdrawn, the rats were killed by severing the aorta. The serums of the samples were separated through centrifuge at 3000 g for fifteen minutes and were kept at a temperature of –80°C for further analyses. After sacrificing the rats, liver tissues were separated and divided into three parts. The first part was frozen in liquid nitrogen for ribonucleic acid (RNA) extraction, the second part was kept at –80°C to produce tissue homogenate for antioxidant parameter evaluation, and the third part was fixed using 10% neutral formalin for histological assessments.

### 2.6. Tissue Homogenization

Lysis buffer was prepared just prior to use. For tissue preparation, 100 mg of liver was homogenized using liquid nitrogen in a mortar. The solution used for homogenization consisted of 10 mM HEPES, 10 mM KCl, 1.5 mM MgCl_2_, 1 mM EDTA, 0.5 mM DTT, and 0.2% Triton X-100. Immediately after, 2 microliters of protease inhibitor were added. The total homogenate was centrifuged at 4° at 10,000 g for 20 min, to obtain the supernatant.

### 2.7. Biochemical Liver Function Tests

The levels of serum aspartate aminotransferase (AST), alanine aminotransferase (ALT), alkaline phosphatase (ALP), lactate dehydrogenase (LDH), and bilirubin were measured using an automatic biochemistry analyzer (Biotecnica BT3000, Italy).

### 2.8. Liver Histology

Liver tissues fixed in 10% formalin were embedded in paraffin and sectioned with a thickness of 5 *μ*m. Sections were stained using eosin-hematoxylin. A histologist who was blind to the study groups assessed histological changes in liver tissues using a light microscope (Nikon, Japan), and the scores of ductular proliferation, fibrosis, inflammation, and necrosis were determined. LF histopathologic scores were as follows: no fibrosis: score 0; portal enhancement with fibrosis: score 1; septal fibrosis: score 2; incomplete nodule formation: score 3; and complete nodule formation: score 4. Ductular proliferation scores were as follows: no ductular proliferation: score 0; limited portal ductular proliferation: score 1; septal ductular proliferation: score 2; incomplete nodule formation: score 3; and complete nodule formation: score 4. Fibrosis scores or staging were determined according to the scheme given by Ishak et al. [23].

### 2.9. Determination of Oxidative Stress Biomarkers

The levels of nitrite and nitrate, as indices for nitric oxide (NO) formation, were measured using Griess reaction and sodium nitrite as standard (0–50 *μ*mol/L). The concentration of reduced glutathione (GSH) in liver tissue homogenate and serum was measured using DTNB which forms a yellow color complex with GSH. Moreover, the MDA of tissue and serum was determined based on the reaction of this compound with TBA, formation of pink color complex, and colorimetric assay. The optical absorption of the complex was measured at a wavelength of 540 nm.

Total antioxidant capacity (TAC) in serum and liver tissue was assessed using the ferric reducing antioxidant power (FRAP) method. In this method, TAC was estimated based on the reduction of a ferric tripyridyltriazine (Fe^3+^-TPTZ) complex to Fe^2+^ form using FeSO_4_.7H_2_O solution as standard (0.1–1 mmol/L).

Protein carbonyl content was measured using the spectrophotometric method based on the color produced in the reaction of carbonyl groups with DNPH. Serum carbonyl level was calculated using a molar absorption coefficient of 2.2 × 10^4^ M^–1^cm^–1^. The level of superoxide dismutase (SOD) enzyme activity in serum and tissue was also measured using an SOD colorimetric assay kit (Kiazist Co., Iran) and reported as U/mg of protein. Moreover, catalase enzyme activity in serum and tissue was measured through the spectrophotometric method based on the reaction between undecomposed hydrogen peroxide and ammonium molybdate and production of a yellowish color. The maximum optical absorption of this color is 374 nm. Enzyme activity was reported as kU/L for serum and kU/mg protein for tissue.

### 2.10. Isolating RNA and Assessing the Expression of the Genes Involved in Liver Inflammation and Fibrogenesis

RNA isolation from liver tissues and cDNA synthesis were performed using the methods reported in a previous study [24]. Then, the relative expression of mRNA was measured using quantitative real-time polymerase chain reaction (PCR) on Bio-Rad MyiQ™ (Bio-Rad Laboratories Inc., Hercules, CA) and was determined using the *ΔΔ*CT method normalized by *β*-actin. Primers were evaluated using the Oligo (v. 7.60) and the Primer-Blast software. The oligonucleotide sequences of the used primers were as follows: 5′-CCTCAGGCTTGGGTCTTGTTA and 5′-CATCCTGTGTTGTTGGGCTG for induced nitric oxide synthase (iNOS); 5′-GGAGCTTGGAACGCGAAGAA and 5′-ACACAAGCCCATTTCAGGGT for caspase-3; 5′-AGGGCTACCATGCCAACTTC and 5′-CCACGTAGTAGACGATGGGC for transforming growth factor beta 1 (TGF*β*-1); 5′-CAGCTATGTGGGGGACGAAG and 5′-TCCGTTAGCAAGGTCGGATG for *α*-smooth muscle actin (*α*-SMA); and 5′-CTGTGTGGATTGGTGGCTCT and 5′-CAGCTCAGTAACAGTCCGCC for *β*-actin.

### 2.11. Statistical Analysis

The one-way analysis of variance and Tukey's post hoc test were used for data analysis. All analyzes were performed using the SPSS (v. 22.0) software. Values were presented as mean ± standard deviation (SD), and the level of significance was set at less than 0.05.

### 2.12. Ethical Considerations

This study has the approval of the Ethics Committee of Kashan University of Medical Sciences, Kashan, Iran (code: IR.KAUMS.MEDNT.REC.1397.114). All procedures were performed under the ethical guidelines for animal care of this university in order to minimize suffering in rats.

## 3. Findings

### 3.1. SS Seed Extract Contains Phenolic Compounds

**Figure 1:**
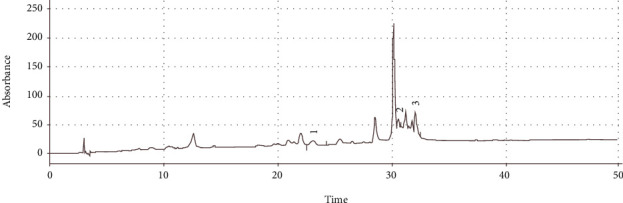
Chromatogram of *Securigera securidaca* extract. Chromatogram of three standard mixtures of phenolic compounds was depicted. The standard phenolic compounds include (1) apigenin 7-glycoside, (2) luteolin, and (3) isorhamnetin. Three phenolic compounds identified in SS hydroalcoholic extract were labeled.

Analysis of the total phenolic and flavonoid content of SS seed extract showed that total phenolic content was 12.09 mg gallic acid equivalent (%*w*/*w*) and total flavonoid content was 0.41 mg QE (%*w*/*w*). SS seed extract was analyzed through HPLC, and the identified peaks were compared with standard values respecting relative retention time (Figure 1). The peaks found in the chromatogram of SS seed extract showed the presence of apigenin 7-glycoside, luteolin, and isorhamnetin with a concentration of, respectively, 1.136, 0.078, and 2.23 mg per gram of the SS seed extract powder.

### 3.2. SS Seed Extract Reduces Liver Function Indices in Rats with BDL

The serum activity levels of liver function enzymes AST, ALT, ALP, and LDH and the levels of total and direct bilirubin in the BDL-control group were significantly greater than the sham, healthy, BDL+SS100, and BDL+SS200 groups (*P* < 0.05; Table 1). These findings show the effectiveness of both 100 mg/kg and 200 mg/kg SS seed extracts in reducing BDL-induced liver injury.

The activity levels of ALT and LDH enzymes and the levels of total and direct bilirubin in both BDL+SS100 and BDL+SS200 groups were significantly lower than those in the BDL+VitC group (*P* < 0.05; Table 1). Moreover, the levels of ALP and AST enzyme activity in the BDL+SS100 group were significantly lower than those in the BDL+VitC and BDL+SS200 groups in a dose-dependent manner (*P* < 0.05; Table 1). These findings imply that SS seed extract, particularly at 100 mg dose, is more effective than 4.25 *μ*g/kg vitamin C in reducing the values of biochemical liver function indices in rats with BDL.

### 3.3. SS Seed Extract Reduces BDL-Induced Oxidative Stress

The levels of oxidative stress biomarkers in serum and liver tissue were measured to assess the mechanism of the protective effects of SS seed extract against BDL-induced liver injury.

#### 3.3.1. Oxidative Stress Biomarkers in Liver Tissue

In the BDL-control group, the levels of NO, MDA, and protein carbonyl were significantly greater, and the levels of TAC, GSH, and SOD and catalase enzyme activity were significantly lower than in the sham group (*P* < 0.05; Table 1). Moreover, the level of nitric oxide metabolite in the BDL+SS100 and the BDL+SS200 groups was significantly lower than that in the BDL-control group by, respectively, 43% and 27% (*P* < 0.05). The level of MDA in the BDL+SS100 group was also significantly lower than the BDL-control group by 41% (*P* < 0.05), while its level in the BDL+SS200 group did not significantly differ from the BDL-control group (*P* > 0.05) (Table 1). The level of protein carbonyl in the BDL+SS100 group did not significantly differ from the BDL-control group (*P* > 0.05), while its level in the BDL+SS200 group was significantly lower than that in the BDL-control group by 15% (*P* < 0.05) (Table 1).

There was no significant difference between the BDL+SS100 and the BDL-control groups respecting the level of TAC (*P* > 0.05), while the level of TAC in the BDL+SS200 group was significantly greater than that in the BDL-group by 23% (*P* < 0.05) (Table 1). Moreover, the levels of GSH as well as SOD and catalase enzyme activity in both BDL+SS100 and BDL+SS200 groups were significantly greater than those in the BDL-control group (*P* < 0.05; Table 1).

Group comparisons respecting oxidative stress biomarkers in liver tissue also showed that the levels of NO, MDA, and protein carbonyl in the BDL+SS100 group were significantly lower than those in the BDL+VitC group, and the levels of GSH as well as SOD and catalase enzyme activity in the BDL+SS100 group were significantly greater than those in the BDL+VitC group (*P* < 0.05; Table 1). Moreover, in the BDL+SS200 group, the level of protein carbonyl was significantly lower than in the BDL+VitC group and the levels of TAC and GSH were significantly greater than in the BDL+VitC group (*P* < 0.05; Table 1). These findings imply that SS seed extract, particularly at 100 mg/kg dose, has greater effectiveness than 4.25 *μ*g/kg vitamin C in reducing oxidative stress and improving antioxidant parameters in a dose-dependent manner.

#### 3.3.2. Oxidative Stress Biomarkers in Serum

In comparison with the sham group, the levels of NO, MDA, and protein carbonyl in the BDL-control group were significantly greater, while the levels of GSH, TAC, and SOD and catalase enzyme activity were significantly lower (*P* < 0.05; Table 1). Moreover, the level of NO metabolite in both BDL+SS100 and BDL+SS200 groups did not significantly differ from the BDL-control group (*P* > 0.05). However, the levels of MDA and protein carbonyl in the BDL+SS100 group were significantly lower than those in the BDL-control group, and the level of MDA in the BDL+SS200 group was significantly lower than that in the BDL-control group (*P* < 0.05). The difference between the BDL+SS200 and BDL-control groups respecting MDA was not significant (*P* > 0.05) (Table 1).

The level of TAC in the BDL+SS200 group was significantly greater than that in the BDL-control group (*P* < 0.05), while the TAC level in the BDL+SS100 group did not significantly differ from that in the BDL-control group (*P* > 0.05). Moreover, the levels of GSH as well as SOD and catalase enzyme activity in both BDL+SS100 and BDL+SS200 groups were significantly greater than those in the BDL-control group (*P* < 0.05) (Table 1).

In comparison with the BDL+VitC group, the levels of MDA and protein carbonyl in the BDL+SS100 group were significantly lower and the levels of GSH as well as SOD and catalase enzyme activity were significantly greater (*P* < 0.05). In the BDL+SS200 group, the level of protein carbonyl was significantly lower than in the BDL+VitC group and the levels of TAC, GSH, as well as SOD and catalase enzyme activity were significantly greater than in the BDL+VitC group (*P* < 0.5) (Table 1).

### 3.4. SS Seed Extract Reduces the Expression of the Genes Involved in BDL-Induced Liver Injury

**Figure 2:**
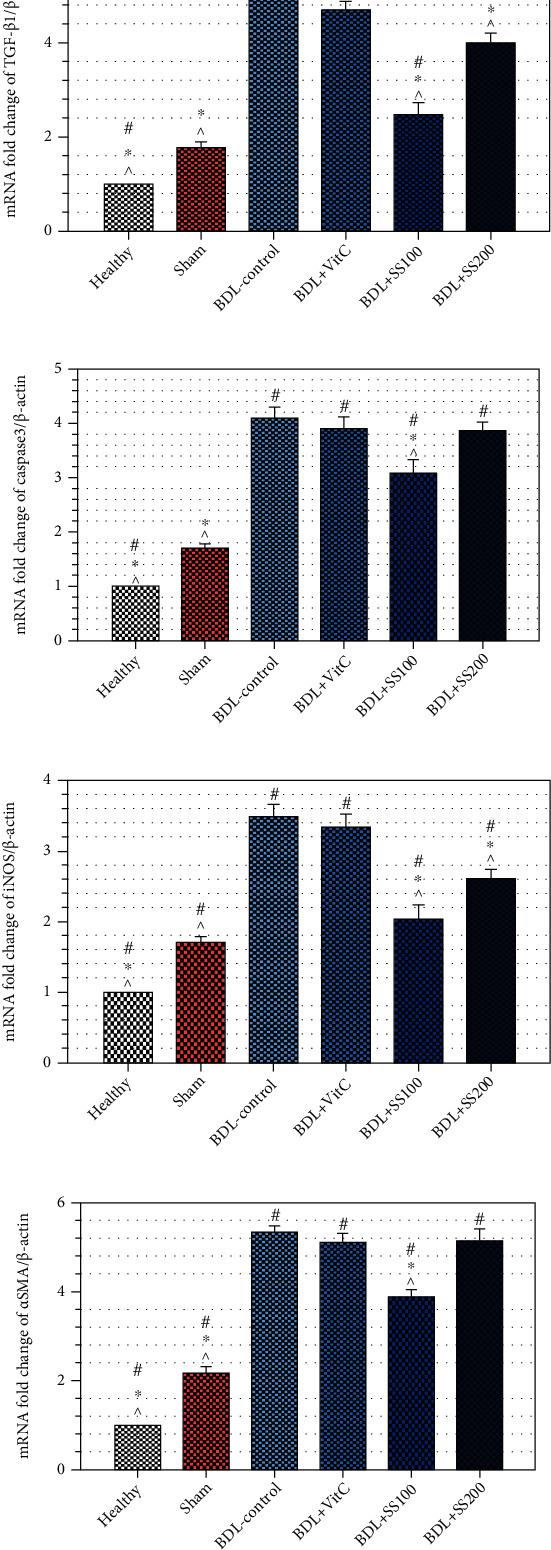
Effects of *Securigera securidaca* seed extract on the gene expression of (a) TGF-*β*1, (b) caspase-3, (c) iNOS, and (d) *α*-SMA. ^#^Significantly different from the sham group; ^∗^significantly different from the BDL-control group; ^^^significantly different from the BDL+VitC group.

The results of quantitative real-time PCR showed that the expression of the TGF*β*-1, iNOS, caspase-3, and alpha-smooth muscle actin (*α*-SMA) genes at mRNA level in the BDL-control group was significantly greater than that in the sham group by 5, 3.5, 4.1, and 5.4 times, respectively (*P* < 0.05). Moreover, the expression of all these genes in the BDL+SS100 group was significantly lower than that in the BDL-control and BDL+SS200 groups in a dose-dependent manner (*P* < 0.05) (Figure 2). These results indicate the positive effects of SS seed extract on BDL-induced liver inflammation in rats.

The expression of the TGF*β*-1, iNOS, caspase-3, and *α*-SMA genes in the BDL+SS100 group was significantly lower than that in the BDL+VitC group. In the BDL+SS200 group, the expression of only the TGF*β*-1 and iNOS genes was significantly lower than in the BDL+VitC group (*P* < 0.05) (Figures 2(a) and 2(c)), and there was no significant difference between these groups in terms of the expression of the caspase-3 and *α*-SMA genes (*P* > 0.05) (Figures 2(b) and 2(d)).

### 3.5. SS Seed Extract Reduces BDL-Induced LF

**Figure 3:**
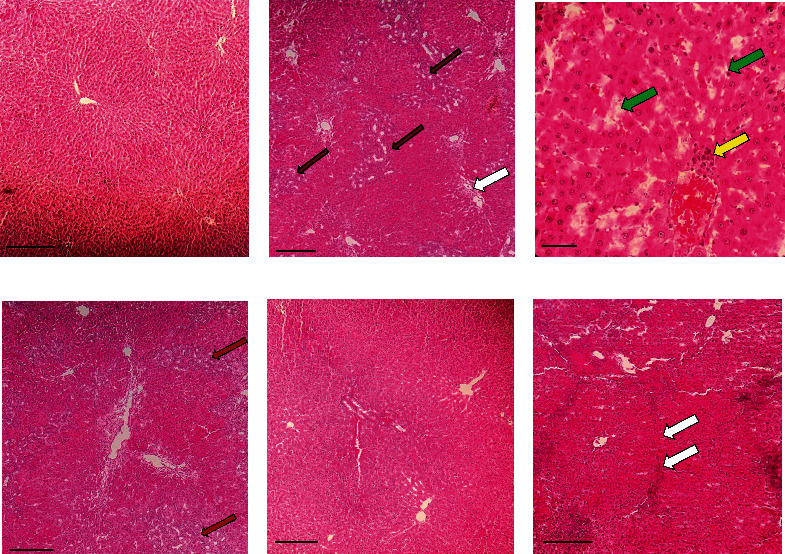
Effects of *Securigera securidaca* seed extract on the histopathological changes of the liver in BDL-afflicted rats. Representative photomicrographs of liver sections processed for H and E staining (10; scale bar 5 mm). (a) Sham X100; (b) BDL-control X100; (c) BDL-control X400; (d) BDL+VitCX100; (e) BDL+SS100 X100; and (f) BDL+SS200 X100. (a) Represents the normal liver histopathology. (b, c) Fibrosis (white arrow) and bile duct hyperplasia (red arrow) in addition to extensive tissue necrosis (green arrow) and inflammation (yellow arrow) are evident in rats with untreated BDL as shown in (b) and (c). In (d) and (c), bile duct hyperplasia is indicated by red arrow, and fibrosis is marked by white arrow. All these lesions markedly decreased in rats treated with 100 mg/kg SS seed extract.

Liver tissues in both healthy and sham groups had normal structure and obtained zero scores for LF-related changes (Table 2). Extensive collagen infiltration, fibrosis, and necrosis together with some levels of inflammation and hyperplasia of bile ducts were observed in rats with untreated BDL. Although rats in the BDL+SS100 and BDL+SS200 groups had some levels of LF, liver morphology and parenchyma structure in the BDL+SS100 group were significantly better than those in the BDL-control and BDL+SS200 groups in a dose-dependent manner (*P* < 0.05) (Table 2; Figure 3).

The levels of fibrosis, inflammation, necrosis, and hyperplasia in bile ducts in the BDL+SS100 and BDL+SS200 groups were significantly lower than those in the BDL+VitC group (*P* < 0.05) (Table 2). These findings indicate that SS seed extract at both doses has greater effectiveness than 4.25 *μ*g/kg vitamin C in preventing LF in rats with BDL.

### 3.6. Mortality and Toxicity

The mortality rate during the 3 weeks of experiment was 25% (12 rats). In the BDL group (*n* = 12), 4 rats died (33.3%); in the BDL+VitC group (*n* = 12), 3 rats died (25%); in the BDL+SS100 group (*n* = 12), 2 rats died (16.6%); and in the BDL+SS200 group (*n* = 12), only 3 rats died (25%) without significant difference between groups at the end of 3 weeks after fibrosis induction. The ethanolic extract of SS is reported to be safe up to a dose of 2 g/kg body weight with no signs of behavioral changes or toxicity observed which suggests its safety.

At the end of the intervention, none of the rats in these groups showed SS side effects such as diarrhea and skin rashes.

## 4. Discussion

Our results showed that SS seed extract is effective in protecting the liver against inflammatory and oxidative damages caused by BDL-induced cholestasis in rats, and its effects are greater than a low dose of ascorbic acid in a dose-dependent manner.

In this study, the hydroalcoholic extract of SS seed was analyzed and compounds such as luteolin, apigenin 7-glycoside, isorhamnetin, gallic acid, and quercetin were successfully identified. Scholars in a study identified 47 phenolic acids and flavonoids in SS flowers [20]. Phenolic acids, like luteolin, apigenin 7-glycoside, isorhamnetin, gallic acid, and quercetin, can reduce inflammation in the tissues of animal models through producing antioxidant and anti-inflammatory effects [22].

Our findings also indicated that SS seed extract at both 100 and 200 mg/kg doses significantly reduced the level of NO metabolite in liver tissue in rats with BDL and the difference between these two doses was not significant. This is probably due to the reduction in the expression of the iNOS gene in liver tissue which encodes the NO synthase enzyme (Figure 2(c)). The level of NO metabolite increases in both serum and liver tissue in rats with BDL [25]. In agreement with our findings, a study reported that treatment with 100, 200, and 400 mg/kg doses of SS seed extract inhibited the increase in NO level in rats with diabetes mellitus [26]. NO is a highly reactive molecule produced in hepatocytes. SS seed extract seems to reduce the reactive species of nitrogen such as NO at intracellular level and thereby reduce oxidative stress and prevent liver injury. Moreover, we found that although NO level significantly decreased in liver tissue, it did not significantly change in serum. This finding is attributable to the fact that NO is highly volatile. A study reported that measurement of tissue NO provides more valuable results compared with serum NO measurement and, hence, generalizing the results of serum or blood NO measurements to some specific tissues is no longer a valid approach [27].

The level of MDA in BDL-afflicted rats was significantly greater than rats in the sham group. Treatment with 100 mg SS seed extract for 21 days significantly reduced the tissue and serum levels of MDA, while treatment with 200 mg SS seed extract produced no significant effects on MDA level. MDA is a marker of tissue injury released from the liver of rats with BDL due to the toxicity of reactive oxygen species. A study reported a significant increase in the level of MDA in rats with BDL-induced cholestasis [28]. In agreement with our findings, some studies found that some antioxidants, such as curcumin and *Phaseolus trilobus*, have protective effects against BDL-induced liver injury [29, 30]. Moreover, a study reported that SS seed extract significantly reduced MDA in rats with diabetes mellitus [21]. It seems that polyphenols with phenolic hydroxyl groups in SS can trap free radicals and reduce lipid peroxidation. Also, high levels of phenols and flavonoids in SS seed extract may reduce protein and lipid peroxidation and increase antioxidant capacity in rats with BDL. In addition, it seems that increased TAC level is related to the presence of antioxidants in SS seed extract.

Interestingly, treatment with 100 mg/kg SS seed extract in the present study was more effective than treatment with 200 mg/kg SS seed extract in increasing the levels of SOD and catalase enzyme activity. Similarly, several studies showed the greater effectiveness of the lower doses of herbal extracts in increasing the activity of antioxidant enzymes. An explanation for this finding may be the toxic effects of the high doses of some extracts [31, 32].

Study findings also revealed that the level of the TGF*β*-1 gene expression in the BDL-control group was significantly greater than the sham and the healthy groups and SS seed extract, particularly at 100 mg/kg dose, and significantly reduced the expression of the TGF*β*-1 and the *α*-SMA genes. Studies on animal models showed that preventing TGF*β*-1 activity is an effective method for inhibiting fibrotic response to liver injury [33]. A study showed that BDL significantly increased profibrogenic cytokines such as TGF*β*-1 and extracellular matrix indices such as *α*-SMA at gene expression level, while treatment with ethyl acetate fraction of *Ammomum xanthoides* significantly reduced them [34]. The pathogenesis of LF includes several mechanisms such as inflammation, growth factor signaling, and lipid signaling. The inflammation pathway and the growth factor signaling pathway mediated by TGF*β*-1 are the most important pathways involved in fibrosis. The fibrogenic cytokine TGF*β* plays significant roles in different physiological processes [33]. Therefore, understanding molecular mechanisms involved in TGF*β* signaling in different diseases is essential to develop effective treatments for them [35, 36].

We also observed a significant increase in the expression of the iNOS gene in BDL-afflicted rats which significantly decreased after treatment with 100 and 200 mg/kg SS seed extracts. A study revealed that NF-*κ*B activation upregulates the transcription of several genes involved in inflammation and apoptosis pathways such as the iNOS gene [37]. Moreover, in oxidative stress, reactive oxygen species can activate gene expression through NF-*κ*B and thereby upregulate the expression of different genes such as iNOS, increase NO production, and start the cascade of apoptosis pathway [24]. This pathway can in turn alter the normal functions of the tissues and cause inflammation, apoptosis, and other liver complications [37]. SS seed extract in the present study might have reduced inflammation and fibrosis through reducing the expression of the iNOS gene and reducing NO production.

We also found that SS seed extract, particularly at 100 mg/kg dose, significantly reduced the expression of the caspase-3 gene in rats with BDL. A study on a carbon tetrachloride-induced LF model showed high levels of the caspase-3 gene expression which reduced after treatment with salvianolic acid [38]. Apoptosis is a proinflammatory process which plays a significant role in LF. Caspase-3 enzyme is the principal enzyme in this process which triggers enzymatic events and leads to cell death.

Another finding of the present study was that both 100 and 200 mg/kg doses of SS seed extract had greater effectiveness than daily supplementation with 4.25 *μ*g/kg vitamin C in rats with BDL. A study revealed that SS seed extract contains ascorbic acid, dodecanedioic acid derivatives, *β*-sitosterol, and oxygenated hydrocarbons such as acyl glucuronides, *α*-D-glucopyranose, N-butylglycine, and 1,3-propanediol [39]. The greater effects of SS seed extract than ascorbic acid in rats with BDL may be due to the presence of ascorbic acid, phenolic compounds, and flavonoids in the extract and their synergetic effects. Phytochemical analysis by Aldal'in et al. showed high content of aromatic derivatives, dodecanedioic acid derivatives and L-ascorbic acid, and *β*-sitosterol and oxygenated hydrocarbons in this plant [40]. Therefore, the higher efficacy of the plant extract as compared to ascorbic acid in BDL rats could be due to the synergistic effect of these compounds (including ascorbic acid) present in the extract.

Overall, our findings indicated that compared with 200 mg/kg SS seed extract, 100 mg/kg SS seed extract had greater effectiveness in reducing BDL-induced cholestatic liver injury in a dose-dependent manner in rats. Histological assessment also confirmed the greater effectiveness of 100 mg/kg SS seed extract in improving the morphology and parenchymal structure of the liver. Similarly, a study on rats reported negative relationship between the dose and the antidiabetic effects of SS seed extract [41]. This finding may be due to the higher viscosity and the lower absorbability of SS seed extract at higher doses. Contrarily, a study reported that different doses of SS seed extract (i.e., 100, 200, and 400 mg/kg) significantly reduced oxidative/nitrosative stress and inflammation in diabetic rats, and the effects of higher doses were greater [26]. This contradiction may be due to the differences between the pathophysiology of DM and cholestatic LF as well as the differences between these studies respecting the daily volume of administered SS seed extract.

## 5. Conclusion

SS seed extract is effective in protecting the liver against inflammatory and oxidative damages caused by BDL-induced cholestasis in rats, and its effects are greater than a low dose of ascorbic acid in a dose-dependent manner. Further studies are still needed to identify active compounds in SS seed extract and the molecular mechanisms of the effects of the extract on liver tissue.

## Figures and Tables

**Table 1:** Group comparisons respecting the mean scores of liver injury biochemical factors and oxidative stress markers.

Markers	Groups					
	Healthy mean ± SD	Sham mean ± SD	BDL-control mean ± SD	BDL+VitC mean ± SD	BDL+SS100 mean ± SD	BDL+SS200 mean ± SD
**Biochemical**						
AST (IU/L)	182.8 ± 9.26^∗^^^^	203.8 ± 15.87^∗^^^^	268.4 ± 11.01^#^	249 ± 13.67^#^	214 ± 6.52^∗^^^^	237.8 ± 8.9^#^^∗^
LT (IU/L)	81.6 ± 7.47^∗^^^^	94.2 ± 7.01^∗^^^^	150.2 ± 8.12^#^^	108.6 ± 7.83^#^^∗^	125.4 ± 7.02^#^^∗^^^^	134.2 ± 6.38^#^^∗^^^^
LP (IU/L)	544.6 ± 36.91^∗^^^^	715.8 ± 69.32^∗^^^^	1316.2 ± 215.11^#^^	1076.4 ± 117.43^#^^∗^	804.6 ± 181.46^∗^^^^	970 ± 46.83^#^^∗^
LDH (IU/L)	2327.8 ± 141.81^∗∧^	2621.2 ± 88.72^∗∧^	4084.8 ± 129.45^#^	3784.2 ± 108.36^#^	3148 ± 150.65^#∗∧^	3466 ± 207.72^#∗∧^
Total bilirubin (mg/dL)	0.3 ± 0^∗∧^	0.4 ± 0^∗∧^	8.08 ± 1.15^#^	6.44 ± 2.37^#^	3.14 ± 0.6^#∗∧^	4.08 ± 0.37^#∗∧^
Direct bilirubin (mg/dL)	0.1 ± 0^∗∧^	0.1 ± 0^∗∧^	6.1 ± 1.14^#^	5.26 ± 0.36^#^	1.58 ± 0.65^#∗∧^	2.17 ± 0.81^#∗∧^
**Oxidative stress markers**						
Liver NO (*μ*mol/mg protein)	0.113 ± 0.02^#∗∧^	0.17 ± 0.01^∗∧^	0.4 ± 0.02^#∧^	0.29 ± 0.01^#∗^	0.228 ± 0.03^#∗∧^	0.29 ± 0.01^#∗^
Liver MDA (*μ*mol/mg protein)	2.8 ± 0.56^∗∧^	3.23 ± 0.45^∗∧^	10.19 ± 0.42^#^	10.72 ± 0.7^#^	6.03 ± 0.33^#∗∧^	10.16 ± 0.39^#^
Liver protein carbonyl (*μ*mol/mg protein)	926.64 ± 23.81^#∗∧^	1023.61 ± 27.75^∗∧^	1224.45 ± 35.03^#^	1201.8 ± 34.86^#^	1174.95 ± 32.81^#∧^	1037.98 ± 23.46^∗∧^
Liver TAC (*μ*mol/mg protein)	1.226 ± 0.05^∗∧^	1.166 ± 0.05^∗∧^	0.728 ± 0.03^#^	0.74 ± 0.04^#^	0.748 ± 0.03^#^	0.846 ± 0.04^#∗∧^
Liver GSH (mmol/mg protein)	0.073 ± 0.001^#∗∧^	0.064 ± 0.011^∗∧^	0.024 ± 0.002^#^	0.028 ± 0.001^#^	0.048 ± 0.003^#∗∧^	0.042 ± 0.002^#∗∧^
Liver SOD (U/mg protein)	6.26 ± 0.67^∗∧^	6.18 ± 0.25^∗∧^	4.73 ± 0.25^#^	5.09 ± 0.28^#^	5.8 ± 0.36^∗∧^	5.41 ± 0.28^#∗^
Liver catalase (U/mg protein)	33.92 ± 2.22^#∗∧^	30.76 ± 1.23^∗∧^	22.1 ± 1.54^#^	24.33 ± 0.58^#^	27.95 ± 1.16^∗∧^	26.32 ± 1.02^#∗^
Serum NO (*μ*mol/L)	0.084 ± 0.02^∗∧^	0.148 ± 0.09^∗∧^	0.346 ± 0.02^#∧^	0.264 ± 0.05^#∗^	0.38 ± 0.02^#∧^	0.302 ± 0.02^#^
Serum MDA (*μ*mol/L)	3.27 ± 0.41^∗∧^	3.72 ± 0.31^∗∧^	11.48 ± 1.12^#^	12 ± 0.59^#^	6.77 ± 1.07^#∗∧^	12.54 ± 0.84^#^
Serum protein carbonyl (*μ*mol/L)	1123.29 ± 61.71^∗∧^	1214.74 ± 59.3^∗∧^	1513.02 ± 31.18^#∧^	1393.33 ± 71.4^#∗^	1195.76 ± 38.84^∗∧^	1134.95 ± 57.94^∗∧^
Serum TAC (*μ*mol/L)	1.30 ± 0.03^#∗∧^	1.25 ± 0.03^∗∧^	0.86 ± 0.03^#^	0.83 ± 0.02^#^	0.87 ± 0.04^#^	0.93 ± 0.02^#∗∧^
Serum GSH (mmol/L)	0.052 ± 0.002^#∗∧^	0.044 ± 0.011^∗∧^	0.013 ± 0.001^#∧^	0.022 ± 0.001^#∗^	0.036 ± 0.002^#∗∧^	0.032 ± 0.002^#∗∧^
Serum SOD (U/mg protein)	2.61 ± 0.22^∗∧^	2.39 ± 0.20^∗∧^	1.4 ± 0.2^#∧^	1.88 ± 0.14^#∗^	2.35 ± 0.22^∗∧^	2.25 ± 0.2^∗^
Serum catalase (kU/L)	16.64 ± 3.08^∗∧^	14.49 ± 1.02^∗∧^	6.41 ± 1.11^#^	8.7 ± 0.83^#^	11.56 ± 1^#∗∧^	9.68 ± 0.67^#∗^

BDL: bile duct ligation; SS: *Securigera securidaca*; VitC: vitamin C; AST: aspartate aminotransferase; ALT: alanine aminotransferase; ALP: alkaline aminotransferase; LDH: lactate dehydrogenase; NO: nitric oxide; MDA: malondialdehyde; TAC: total antioxidant capacity; GSH: glutathione; SOD: superoxide dismutase. ^#^Significantly different from the sham group; ^∗^significantly different from the BDL-control group; ^^^significantly different from the BDL+VitC group.

**Table 2:** Group comparisons respecting the mean scores of BDL-induced liver injury.

Group	Parameters			
	Fibrosis mean ± SD	Inflammation mean ± SD	Duct hyperplasia mean ± SD	Necrosis mean ± SD
Healthy	0 ± 0	0 ± 0	0 ± 0	0 ± 0
Sham	0 ± 0	0 ± 0	0 ± 0	0 ± 0
BDL-control	3 ± 0.71^##^	2.6 ± 0.55^##^	3 ± 0.71^##^	3.4 ± 0.55^##^
BDL+VitC	2 ± 0.71	3.6 ± 0.55	3.4 ± 0.89	3.2 ± 0.84
BDL+SS100	1.4 ± 0.55^∗∗^	0.8 ± 0.45^∗∗^	1.4 ± 0.55^∗^	0.8 ± 0.45^∗∗^
BDL+SS200	2.4 ± 0.55	2 ± 1	2.8 ± 0.84	2.2 ± 0.45^∗^

BDL: bile duct ligation; SS: *Securigera securidaca*; VitC: vitamin C; ^#^significantly different from the sham group; ^∗^significantly different from the BDL-control group; ^^^significantly different from the BDL+VitC group.

## Data Availability

Data are available on request.
